# Randomized trial of achieving healthy lifestyles in psychiatric rehabilitation: the ACHIEVE trial

**DOI:** 10.1186/1471-244X-10-108

**Published:** 2010-12-13

**Authors:** Sarah S Casagrande, Gerald J Jerome, Arlene T Dalcin, Faith B Dickerson, Cheryl A Anderson, Lawrence J Appel, Jeanne Charleston, Rosa M Crum, Deborah R Young, Eliseo Guallar, Kevin D Frick, Richard W Goldberg, Meghan Oefinger, Joseph Finkelstein, Joseph V Gennusa, Oladapo Fred-Omojole, Leslie M Campbell, Nae-Yuh Wang, Gail L Daumit

**Affiliations:** 1 Welch Center for Prevention, Epidemiology, and Clinical Research, Johns Hopkins University, Baltimore, Maryland, USA; 2 Department of Medicine, Johns Hopkins University School of Medicine, Baltimore, Maryland, USA; 3 Department of Kinesiology, Towson University, Towson, Maryland, USA; 4 Sheppard Pratt Health System, Towson, Maryland, USA; 5 Department of Epidemiology, Johns Hopkins Bloomberg School of Public Health, Baltimore, Maryland, USA; 6 Department of Health Policy and Management, Johns Hopkins Bloomberg School of Public Health, Baltimore, Maryland, USA; 7 VA Capitol Health Care Network (VISN 5) Mental Illness, Research, Education and Clinical Center, Baltimore, Maryland, USA; 8 Department of Psychiatry, University of Maryland School of Medicine, Baltimore, Maryland, USA; 9 Department of Epidemiology and Biostatistics, University of Maryland School of Public Health, College Park, Maryland, USA; 10 Department of Psychiatry, Johns Hopkins University School of Medicine, Baltimore, Maryland, USA; 11 Department of Mental Health, Johns Hopkins Bloomberg School of Public Health, Baltimore, Maryland, USA

## Abstract

**Background:**

Overweight and obesity are highly prevalent among persons with serious mental illness. These conditions likely contribute to premature cardiovascular disease and a 20 to 30 percent shortened life expectancy in this vulnerable population. Persons with serious mental illness need effective, appropriately tailored behavioral interventions to achieve and maintain weight loss. Psychiatric rehabilitation day programs provide logical intervention settings because mental health consumers often attend regularly and exercise can take place on-site. This paper describes the Randomized Trial of Achieving Healthy Lifestyles in Psychiatric Rehabilitation (ACHIEVE). The goal of the study is to determine the effectiveness of a behavioral weight loss intervention among persons with serious mental illness that attend psychiatric rehabilitation programs. Participants randomized to the intervention arm of the study are hypothesized to have greater weight loss than the control group.

**Methods/Design:**

A targeted 320 men and women with serious mental illness and overweight or obesity (body mass index ≥ 25.0 kg/m^2^) will be recruited from 10 psychiatric rehabilitation programs across Maryland. The core design is a randomized, two-arm, parallel, multi-site clinical trial to compare the effectiveness of an 18-month behavioral weight loss intervention to usual care. Active intervention participants receive weight management sessions and physical activity classes on-site led by study interventionists. The intervention incorporates cognitive adaptations for persons with serious mental illness attending psychiatric rehabilitation programs. The initial intensive intervention period is six months, followed by a twelve-month maintenance period in which trained rehabilitation program staff assume responsibility for delivering parts of the intervention. Primary outcomes are weight loss at six and 18 months.

**Discussion:**

Evidence-based approaches to the high burden of obesity and cardiovascular disease risk in person with serious mental illness are urgently needed. The ACHIEVE Trial is tailored to persons with serious mental illness in community settings. This multi-site randomized clinical trial will provide a rigorous evaluation of a practical behavioral intervention designed to accomplish and sustain weight loss in persons with serious mental illness.

**Trial Registration:**

Clinical Trials.gov NCT00902694

## Background

The prevalence of overweight and obesity has significantly increased over the past several decades with roughly 34% of U.S. adults currently being obese [1,2]. In persons with serious mental illness (e.g., schizophrenia, bipolar disorder), overweight and obesity are at epidemic levels that are higher than in the overall U.S. population [3-5]. Obesity significantly increases the risk of morbidity and mortality from cardiovascular disease mainly through the effects of obesity on hypertension, hyperlipidemia and diabetes mellitus [6,7]. Furthermore, many classes of psychotropic drugs are associated with weight gain. [3,8,9]. Given the long-term needs of most patients with serious mental illness to continue psychotropic medications, interventions to reduce obesity and cardiovascular risk are urgently needed.

National guidelines for weight loss emphasize a multifaceted approach to include reduced energy intake, improved dietary patterns and increased physical activity [10-13]. Randomized controlled behavioral weight loss intervention trials showing efficacy for weight loss in the general population have systematically excluded individuals with chronic mental illness from participating [14-19]. In addition, behavioral lifestyle interventions in persons with serious mental illness pose several challenges since these individuals may have competing demands on a daily basis. Person with serious mental illness are often dealing with active mental health symptoms, housing issues, substance abuse or other social issues that may take precedence over behavioral change for weight loss. Furthermore, the high prevalence of cognitive deficits in this population can impede individuals from successfully performing activities of daily living [20]. Thus, traditional behavioral interventions that have been shown to be effective in populations without mental illness should be tailored to meet the specific needs of persons with serious mental illness [14].

There have been few behavioral interventions that have targeted weight loss in persons with serious mental illness. Most previous studies have applied strategies similar to those used in trials for the general population and have focused exclusively on dietary intake with a few incorporating some physical activity component. Furthermore, the interventions in this population have been small (< 60 participants), without a control comparison group and have used various methods to induce behavior change [21-28]. Nevertheless, the literature indicates that selected persons with serious mental illness can benefit from short-term behavioral weight loss interventions [17]. Given the health benefits of exercise and healthy dietary patterns, randomized controlled trials of comprehensive weight loss interventions (dietary and physical activity components) are needed among persons with serious mental illness.

This protocol describes a randomized, controlled trial of a comprehensive behavioral weight loss and weight maintenance program among persons with serious mental illness that attend psychiatric rehabilitation day programs across Maryland. Psychiatric rehabilitation programs (PRP) are outpatient facilities where persons with serious mental illness attend up to several days a week and receive vocational and other services. Psychiatric rehabilitation programs may be opportune and efficient settings for testing, implementing and disseminating interventions. Rehabilitation programs have classroom and communal space suitable for group weight management and exercise sessions. The potential sustainability of a weight loss intervention at psychiatric rehabilitation programs may be more feasible than in other types of mental health settings because consumers regularly attend and the centers already offer other types of classes. In addition, most rehabilitation programs serve persons with a range of serious mental illness diagnoses, including schizophrenia; thus, interventions targeted to these settings could generalize to broad populations with serious mental illness.

## Methods

### Study Design Summary

The core design is a randomized, two-arm, parallel, multi-site clinical trial. Inclusion/exclusion criteria were chosen to enroll a population with chronic mental illness who are overweight or obese and who may safely participate in a weight loss intervention including moderate intensity exercise. Interested mental health consumers are being screened for eligibility at 10 psychiatric rehabilitation centers across Maryland. Three hundred and twenty adult participants completing screening are targeted for enrollment and randomization to the ACHIEVE (Achieving Healthy Lifestyles in Psychiatric Rehabilitation) intervention or usual care. Intervention participants receive group and individual weight management sessions and group physical activity classes. In the initial 6-month intervention phase, study interventionists lead the sessions and also train designated rehabilitation program staff. A 12-month maintenance intervention phase follows in which interventionists continue leading some sessions and trained rehabilitation staff assume responsibility for delivering part of the intervention. Interventionists also provide education to rehabilitation program kitchen staff to increase options for healthy meals served on-site. Follow-up data collection occurs at 6, 12, and 18 months from baseline. The study was approved by the Johns Hopkins Institutional Review Board (protocol number NA00015231).

### Specific Aims

#### Primary Aim

Determine the effect of the ACHIEVE intervention on weight loss at 6 and 18 months. The hypothesis is that the ACHIEVE intervention group will have greater weight loss than the control group.

#### Secondary Aims

Determine the effect of the ACHIEVE intervention on the following outcomes at 6 and 18 months: physical fitness by submaximal bicycle ergometer; waist circumference; blood pressure; lipids; Framingham cardiovascular risk score; health status with SF-12 and depression with CES-D. The hypothesis is that the ACHIEVE intervention group will have greater improvement of these outcomes compared to the control group. In addition, costs per participant will be assessed and a cost effectiveness analysis will be performed.

### Study sites and population

The study sites will include 10 community psychiatric rehabilitation programs across urban and suburban Maryland. Each location shares certain features: (1) adult psychiatric rehabilitation day program with consumers attending regularly at least 3 days a week; (2) space for on-site physical activity classes; and (3) meals served to consumers. The trial is enrolling men and women, age 18 and older who are overweight (body mass index ≥ 25.0 kg/m^2^) or obese and attend the psychiatric rehabilitation programs. By definition of their psychiatric rehabilitation attendance, these participants have a serious mental illness diagnosis. Persons with serious mental illness that receive care from rehabilitation programs' on-site mental health clinics are also eligible for recruitment. The inclusion and exclusion criteria for the trial are listed below.

#### Inclusion criteria

■ Age 18 and older;

■ Overweight, defined by Body Mass Index at least 25.0 kg/m^2^;

■ Able and willing to give informed consent and participate in the intervention;

■ On the same psychiatric medications within the 30 days before baseline weight (dose changes allowed;)

■ Able to attend at least 2 intervention sessions per week (one weight management and one physical activity session) during initial 6-month phase;

#### Exclusion criteria

■ Contraindication to weight loss

- Receiving active cancer treatment (radiation/chemotherapy)

- Liver failure

- History of anorexia nervosa;

■ Cardiovascular event (unstable angina, myocardial infarction) within previous 6 months;

■ Prior or planned bariatric surgery;

Use of prescription weight loss medication or over-the-counter orlistat within 3 months

if participant does not agree to stop taking it;

■ Twenty pound or greater weight loss in 3 months prior to baseline, as documented by staff measurement;

■ Inability to walk to participate in exercise class;

■ Pregnant or planning a pregnancy during study period. Nursing mothers would need approval from physician;

■ Alcohol or substance use disorder either: 1) active and determined to be incompatible with participation in the intervention through discussion with program staff; or 2) new abstinence from alcohol or substance use disorder in past 30 days;

■ Planning to leave rehabilitation center within 6 months or move out of geographic area within 18 months;

■ Investigator judgment (e.g., for concerns over safety, adherence or follow-up);

■ Weight greater than 400 pounds.

### Recruitment Strategies

Most recruitment activities occur on-site at the psychiatric rehabilitation centers and affiliated psychiatric clinics. The primary means of recruitment is direct communication with consumers. Regular mental health consumer meetings at the rehabilitation programs provide opportunities for study staff to present the study. During presentations, study staff often show an informational video and may incorporate an exercise class demonstration. Posters are displayed and fliers and brochures are distributed. In addition, study staff make presentations to rehabilitation program staff and outpatient mental health clinic staff. Rehabilitation program staff and outpatient clinic staff may mention the study to their clients. Potential participants also are identified by working with rehabilitation staff and reviewing together the list of rehabilitation program attendees, using a HIPAA waiver. Study staff are available on-site at the rehabilitation programs to discuss the study during the times when consumers attend. Potential participants are also given a phone number and a postcard to mail back to reach study staff to indicate their interest.

To track recruitment, a roster of consumers attending the center is provided by each rehabilitation program. These rosters assist in defining and counting the number of consumers contacted and enable the characterization of enrollees and non-enrollees.

### Data Collection and Measurements

Before formal screening for the trial begins, oral consent from rehabilitation center attendees is obtained and their weight and height at study baseline is measured for calculation of body mass index; weight will be measured again at 6 and 18-month follow up data collection points. Measuring weight of all consumers at the day program, regardless of their interest in joining the weight loss intervention study, allows for an understanding of: (1) the natural history of weight gain in this population without intervention and (2) how the environmental component of the intervention (i.e., increasing options for healthy meals on-site) may affect weight at the centers in a pre/post fashion. The goal will be to measure height and weight for as many attendees as possible.

For the formal trial, all participants provide written informed consent using procedures reviewed and approved by Johns Hopkins Institutional Review Boards (IRB). A two-stage consent process is used with the first consent obtained to conduct screening procedures and a second consent obtained after baseline data collection and prior to randomization. An evaluation of ability to give consent is also administered for each participant before screening consent which includes answering questions about the goal of the study, what they will be asked to do, and what risks may be involved if they join the study. If a participant is deemed not able to give consent, he/she may not join the study.

Participant eligibility is determined by the completion of several screening measures. The Rose Angina Questionnaire and a checklist of medical conditions are implemented to determine health status for a moderate intensity exercise program [29]. If potential participants have a positive Rose Questionnaire, a prior history of cardiovascular disease or have diabetes mellitus, they require an approval letter from their primary care physician in order to enroll in the study. In addition, primary care physicians for all participants are contacted about the study to ensure there are no contraindications to weight loss or participation in moderate intensity physical activity. Alcohol and substance abuse are assessed with the Addiction Severity Index-Lite [30]; medications are reviewed; and plans to remain at the rehabilitation center during the duration of the study to participate in the trial are discussed with the consumer.

Data collection visits occur on-site at psychiatric rehabilitation centers and are conducted by trained data collectors certified in study measures. Measurements are conducted using standardized operating procedures and quality control methods. Table 1 summarizes the data collection measures and schedule. Follow-up measures are collected at 6, 12, and 18 months after baseline.

**Table 1:** ACHIEVE Trial Data Collection Schedule

	Baseline	6 mo	12 mo	18 mo
**For all rehabilitation enrollees:**				

Age, gender, race	X			

Height	X			

Weight	X	X		X

				

**For trial participants:**				

Sociodemographics (age, gender, race, ethnicity, marital status, education, employment, living arrangements)	X	X		X

Medical history				

Rose Angina Questionnaire [29]	X	X	X	X

Medical conditions	X	X		X

Substance Use (Addiction Severity Index-Lite)[30]	X			

Mental health diagnoses (chart review)	X			

Medications	X	X	X	X

Physical measures				

Height	X			

Weight	X	X	X	X

Submaximal bicycle ergometer	X	X		X

Waist circumference	X	X		X

Blood pressure-3 measures at each of 3 visits, 1 week apart	X	X		X

Fasting serum measures				

Glucose	X	X		X

Insulin	X	X		X

Lipids (total cholesterol, LDL, HDL, triglycerides)	X	X		X

C-reactive protein	X	X		X

Health behaviors				

Block Diet Screeners [57,58]	X	X		X

Direct observation of diet at rehabilitation centers	X	X		X

Food preparation and shopping habits	X	X		X

Physical Activity (International Physical Activity Questionnaire) [59]	X	X		X

Tobacco smoking	X	X		X

Mental health symptoms				

Behavior and Symptom Identification Scale-24 [60]	X	X		X

Center for Epidemiologic Studies Depression Scale [61]	X	X		X

Health status-SF-12 [62]				

Quality of life measures	X	X		X

Euroqol EQ-D [63]				

Impact of Weight on Quality of Life-Lite [64]				

Social support measures				

MOS Social Support Survey [65]				

Social Support and Eating/Exercise (adapted) [66]				

Self-efficacy measures				

General Self Efficacy Scale [67]				

Weight Efficacy Lifestyle Questionnaire [68]				

Physical Activity Barriers Self-Efficacy Scale [69]				

Other measures (binge eating, weight loss history, neighborhood characteristics, mobility, sleep quality) [70-73]				

Safety measures		X	X	X

### Randomization and Blinding

After baseline data collection and before the intervention begins, participants meet with study staff and the intervention arms of the study are described in detail. The study coordinator confirms that the participant meets eligibility criteria, and that all required baseline data have been collected. Participants interested in enrollment are consented individually and randomized to either the intervention or the control group. The study coordinator ascertains and communicates treatment assignments to participants [31]. Randomization is stratified by gender and site using blocks of sizes 2 and 4 in random order to create the randomization sequence for each stratum.

Due to the nature of the intervention, both participants and interventionists will be aware of the assignment. The following mechanisms are in place for data collection staff to be masked to treatment assignment: 1) designating and tracking unmasked study staff; 2) excluding data collection staff from any part of intervention delivery; 3) performing outcome assessments in separate rooms than the intervention; and 4) reminding participants not to share their group assignment. Until the trial end, investigators, staff and participants are masked to outcome data with the exception of the trial statistician and data analyst. In addition, the primary outcome variable, weight, is subject to very little measurement bias [32].

### Intervention

The ACHIEVE intervention incorporates concepts from social cognitive theory, behavioral self-management and the relapse prevention model [33-36]. The theoretical base of the ACHIEVE Trial fits well within the psychiatric rehabilitation framework which emphasizes tenets of intrinsic skills building and environmental supports [37,38]. Motivational interviewing provides an important framework for helping participants problem solve and set goals for weight loss.

The ACHIEVE intervention operationalizes these models by providing frequent and extended contacts, opportunities for group interactions and social support, goal setting and self-negotiation, problem solving, and examples of new behavioral options. The intervention was developed from a comprehensive lifestyle intervention tested in the NHLBI sponsored PREMIER Trial (A Trial of Lifestyle Interventions for Blood Pressure Control), which has proven efficacious for weight loss by incorporating dietary and physical activity components [16,19,39]. To the PREMIER foundation of individual and group counseling sessions, on-site physical activity sessions were added to take advantage of the psychiatric rehabilitation environment as an opportunity for both skills modeling and attaining most of the weekly recommended physical activity. To further tailor the intervention, the neurocognitive deficits in working memory, verbal memory and executive function that are common in persons with serious mental illness were addressed [40,41]. Successful didactic interventions in schizophrenia emphasize learning a few, specific and narrow skills repeatedly, breaking material into small units, learning aides to reduce requirements on memory and attention, repetition of content, and rehearsing behavioral skills [42]. One successful approach for coping with cognitive deficits is the use of compensatory environmental strategies, which are adaptations in the environment designed to bypass neurocognitive impairments and improve adaptive functioning [43]; examples include signs, labels, and devices designed to cue and sequence appropriate behaviors and structure [44]. All aspects of the ACHIEVE intervention were tailored to meet the specific needs of a psychiatric rehabilitation population. Table 2 outlines the cognitive adaptations in the intervention.

**Table 2:** Cognitive adaptations in the ACHIEVE intervention

Self-monitoring	-Tracker: participants mark fruits/vegetables, sugar drinks, junk food, smart portions, smart snacks and exercise. Detailed food and calorie log not required so complexity of recording is simplified. -Weigh-in one time per week during the intensive phase, and once per month during the maintenance phase. Frequent weigh-ins provide opportunity for reinforcement and repetition.
Group weight-management sessions	-Highly structured, emphasis on behavioral rehearsal. -Material taught in small content units. Frequent meetings (1 session per week during the intensive phase, 1 session per month during the maintenance phase) allow repetition of concepts. -Program materials are written at 5^th^-8^th ^grade reading level. -Hands-on activities emphasized. Taste testing, label reading, portion measuring. - Role-playing emphasized. Practicing saying no to junk food, or choosing smarter portions at a birthday party. Increases self-efficacy to adhere to healthier eating habits. -Worksheets review topic of the week.

Individual sessions	-Allow for individualized cognitive tailoring as needed. -Opportunity to emphasize an individualized high impact behavior based upon the concepts learned in groups.

Physical activity classes	-Provide opportunity for modeling and building physical activity skills in supportive setting to increase cardiovascular fitness and exercise self efficacy

Environmental prompts	-Refrigerator magnets, preprinted grocery lists, watches, water bottle, measuring cups, lunch bag as reminders to be used at home.

Reinforcements	-Participation is rewarded with varying levels of gifts relative to the number of classes attended.

The main intervention goals of ACHIEVE include: (1) reducing caloric intake by avoiding sugar drinks and "junk food," (2) eating 5 fruits and vegetables a day, (3) choosing smart portions and snacks, and (4) increasing caloric expenditure through participation in 3 moderate intensity aerobic exercise sessions per week at the psychiatric rehabilitation program [10].

Table 3 reflects the ACHIEVE intervention characteristics for participants randomized to the intervention group. In the first individual session, the interventionist begins a partnership with the participant and assesses his/her readiness to change and understanding nutritional principles. Behavior goals are set and in subsequent sessions the interventionist uses feedback and motivational interviewing techniques to assess the participant's current progress and to help move towards the next goal. These sessions allow the interventionist to tailor the intervention to individual needs.

**Table 3:** Description of ACHIEVE Intervention

Initial Intervention (Months 1 through 6)		
**Type of contacts**	**Frequency**	

**Group weight-management class **led by interventionist (45 min.)	Once per week 3 of 4 weeks	

**Individual visit **with interventionist (15-20 min.)	Once per month	

**Group physical activity class **led by exercise leader/interventionist (50 min.)	Three times per week	

**Weigh-in **during weight management group and individual visit (2 min.)	Once per week	

**Maintenance Intervention (Months 7 through 12)**		

**Type of contacts**	**Frequency**	

	**Months 7 through 12**	**Months 13 through 18**

**Group weight management class** Overall frequency	Once per month	Once per month

Led by interventionist	Monthly	Monthly

**Individual visit **with interventionist	Every 4 weeks	Every 4 weeks

**Group physical activity class** Overall frequency	Three times per week	Three times per week

Led by exercise leader/interventionist	Twice per week	Once per week

Monitored by rehab program staff	Once per week	Twice per week

**Weigh-in** Overall frequency	Twice per month	Twice per month

With intervention staff	Twice per month	Twice per month

**Core Components**		

Self-monitoring	Weigh-ins, Tracker food/exercise log	

Goal setting, feedback, problem solving	Motivational interviewing and support at group and individual sessions	

Social support	Group and individual weight management sessions, group physical activity sessions	

Skills training	Weight management group sessions, physical activity sessions, individual sessions	

Environmental supports	Physical activity sessions, Staff education in health food choices on-site	

Environmental contingencies/reinforcements	incentive items for attendance, participation and specific behaviors	

Group weight-management classes occur three times per month during the intensive phase of the intervention and each month the classes are focused on one main topic, such as increasing fruit and vegetable consumption. The sessions begin with a discussion to support and review the concepts discussed and practiced since the prior session. A portion of the weekly session is devoted to didactic information about healthy eating or physical activity education, which is supported by food models and posters, a self-monitoring worksheet, hands-on activities related to the monthly topic, or food tastings. Participants set individual behavioral goals based on the material presented that week.

Group physical activity sessions are held three days per week at the rehabilitation center (e.g., in a multipurpose area) and led by a trained exercise leader from the study staff. A progressive exercise program starts at a level appropriate for sedentary adults: 10-minute warm-up; 10 minutes of moderate intensity physical activity and 5-minute cool down [45]. The exercise duration gradually increases each week until participants are completing 40 minutes of moderate intensity physical activity and 10 minutes of warm-up and cool-down. Participants are encouraged to incorporate daily physical activity outside of the group exercise class and may set goals that reflect this effort.

In addition to the organized sessions, participants meet with the intervention staff monthly for an individual weight loss counseling session. This brief activity provides immediate feedback on weight loss progress. If participants lose weight, they are asked what worked for them. If they gain weight, staff work to problem-solve and assist in working towards a behavioral goal or setting a more realistic goal.

Self-monitoring and positive reinforcement are important aspects of successful weight loss trials. Participants are asked to fill out a "Tracker" as a self-monitoring tool outside of group sessions. Each tracker is used for one week; participants record the number of servings of fruits and vegetables, and respond yes or no to: exercising for 30 minutes; drinking sugar drinks; eating junk food; smart portions or smart snacks. The Tracker provides a behavioral cue to participants. An incentive program rewards participation in class and individual sessions with choices of varying priced items (e.g., gym suit, store gift card) after a specified number of stickers have been earned. In order to earn a sticker in exercise class the participant must remain standing and engaged in class from the first minute of warm up through the last minute of cool down. For weight management group and individual visits, participants earn a sticker for being present in class throughout the entire duration of the session time.

For the maintenance intervention period, rehabilitation center staff assumes the responsibility for much of the exercise portion of the intervention in a stepped process over two 6-month phases. Designated rehabilitation center employees are trained by intervention study staff and provided with exercise videos made by the study team in an effort to mimic the instructor led exercise class as much as possible. The rehabilitation staff take responsibility for encouraging attendance and participation, starting the video, overseeing the safety of the class, and recording attendance data. Intervention study staff are available for consultation as needed to offer more support during this phase. This transition occurs in order to facilitate the rehabilitation center's ownership of the program, with the goal of increasing the likelihood that the center will continue to offer components of the intervention after the study is complete.

### Intervention Delivery

ACHIEVE interventionists are skilled facilitators with experience in behavior change and group and individual-level counseling. Interventionists have a skill level that would be typical for a community health educator with a bachelor's degree; exercise leaders have at least one year of experience in leading an exercise class and/or are a certified exercise instructor. Intervention staff are trained to deliver any or all components of the intervention in order to maximize the resources of this multi-site trial. Manualized procedures and standardized materials are used to ensure consistency of the intervention including standardized formats for the group exercise classes. Staff members are regularly observed as part of ongoing staff training and fidelity assurance.

Psychiatric rehabilitation program leadership and staff at each site support the intervention and are involved in the study on multiple levels. Program leadership from each site work with the study team so that intervention classes fit into the overall center schedule, and collaborate on participants' individual rehabilitation plans. Each site designates at least one employee to become trained to conduct group physical activity classes using an exercise video.

Resources included in measuring the costs of the intervention delivery include the number of staff, and the duration of each activity. For each study site, staff record one week of data at intervention months 2, 3, 5, 6, 12, 15 and 18. Staff time includes time with participants for intervention sessions, time spent preparing for sessions, training and intervention-related meetings. All research related activities are excluded from the cost analysis.

### Control Group

Participants in the control group receive standard nutrition and physical activity written information at baseline. Health classes are held quarterly for control participants with content unrelated to weight loss (e.g., cancer screening, oral health).

### Environmental Nutrition Intervention

In order to support intervention group participants' ability to select healthy foods, interventionists provide consulting services to rehabilitation program kitchen staff. The consultation sessions help kitchen staff identify healthier food choices for meals served on-site within site budget and regulatory constraints (e.g., federal food guidelines). Interventionists work with rehabilitation staff to identify goals and then offer options to improve food choices such reducing high sugar foods, working on appropriate portion sizes and modifying vending machine offerings. Rehabilitation staff choose goals and which options they will incorporate.

A random sample of menus at baseline, 6 months, and 18 months are collected and evaluated for nutritional content using ESHA software (The Food Processor, 2009, Salem, OR) [46].

### Data Analysis

Randomly assigned intervention group (i.e., behavioral intervention or control) is the main independent variable for intent-to-treat analysis [47]. The co-primary outcomes are weight loss at 6 and 18 months. To evaluate the efficacy of the intervention for each of these outcomes, generalized estimating equations are used [48]. These models account for the longitudinal nature of the trial and incorporate baseline, and 6, 12 and 18-month measurements and will account for study site and other baseline characteristics found not to be balanced by randomization. For secondary outcomes that are categorical, logistic regression GEE models are used according to the same principles outlined above for continuous outcomes.

In addition to the analyses that preserve the intention to treat principle, analyses on subgroups defined post-randomization are exploratory. These include analyses in participants who attended the majority of weight management and exercise intervention sessions.

Although second generation antipsychotics and other psychotropic medications can induce substantial weight gain [3,9], we expect that both study groups will be equivalent in their distribution of these medication due to the process of randomization. Several analytical approaches are planned to address three main potential effects of antipsychotic and other concomitant medication on study outcomes; these include: (1) imbalances in medication use between the intervention and control groups, despite randomization procedures, (2) variation in intervention efficacy by medication or medication class, and (3) changes in drug use after randomization.

The analytic approach to handle missing data will be anchored on the assumption that data is missing at random (MAR), where the probability of missing can depend on all observed information such as measured weights and covariates but does not depend on variables that are not recorded. The analysis model will include parameters for visit specific means for each treatment group, baseline covariates associated with study retention, and use an unstructured covariance structure. Missing at random is almost never strictly correct, but careful modeling should make the missing data process as close to MAR as possible. Primary analyses will be conducted under the assumption of MAR; sensitivity analyses will be based on sensible "missing not at random" scenarios to evaluate the robustness of the inferences under the MAR assumption.

For the environmental nutrition intervention, the menus are collected at each data collection time point and analyzed for nutrients using ESHA software. The mean number of calories, macronutrients, and micronutrients at each site are determined and t-tests are used to determine significant differences at each time point. Given the expected variability between sites in making changes to menus, differences in menu changes will be assessed within each site and then overall.

To support the long-term goal to integrate the ACHIEVE program into psychiatric rehabilitation centers, a cost analysis is planned. The primary analysis assesses the direct cost per participant of intervention implementation from the perspective of a future payer (e.g., Medicaid). A second analysis assesses costs from the societal perspective projecting cardiovascular risk factor changes 10 years into the future.

### Sample size and power

The main objective of this trial is to detect weight loss having public health significance. Previous work has indicated that 4-5 pounds of weight loss should reduce systolic blood pressure by ~3 mmHg, which has been estimated to reduce stroke mortality by 6-8%; cardiovascular heart disease mortality by 4-5%; and to reduce risk of incident hypertension by 20% [18,49]. A Monte Carlo simulation study was used to assess the power to detect a clinically meaningful effect on weight loss at months 6 and 18 under a range of conservative assumptions about the effect size, standard deviation, and follow-up with potentially clustered sites [50]. It was assumed that a 4.5 lb difference in weight at 18 months between intervention and control groups would be observed and that the difference at 6 months would be larger. For power calculations, we assumed a standard deviation of change in weight of 12 lbs and that follow-up would be 80% complete. Under these assumptions, for two-sided 0.05-level tests of the null hypothesis, the study should provide approximately 86% power for detecting a difference of 4.5 lbs with SD = 12 lbs. In addition, the study will have the same power to detect the a similar effect size with a smaller sample size if we achieve a higher follow-up rate.

## Discussion

Despite successful behavioral weight loss interventions in the general population, few randomized controlled trials of comprehensive behavioral weight loss interventions among persons with serious mental illness have been performed [51]. Given the high prevalence of obesity and cardiovascular risk factors, effective weight loss programs are needed in this vulnerable population. The ACHIEVE investigators led a previous pilot weight loss study (n = 52) in two psychiatric rehabilitation programs and demonstrated preliminary success with high levels of recruitment, retention and pre/post weight loss of 4.8 pounds [52]. The ACHIEVE Trial will definitively test the effectiveness of this innovative, practical intervention to realize and sustain weight loss in overweight and obese persons with serious mental illness. If successful, the intervention will be a model program that should provide important health benefits by reducing cardiovascular disease risk for persons with serious mental illness, and with appropriate resources, could be disseminated widely.

This study compares the effectiveness of a multifaceted weight loss intervention to a standard care group among persons who often have cognitive impairments and other comorbidities. Behavioral weight loss trials have shown efficacy for weight loss in other populations. For example, the ACHIEVE intervention was modeled after the PREMIER Trial, a comprehensive lifestyle intervention that incorporated education and counseling for diet and physical activity; the trial was proven effective for weight loss in the general population [39]. The Trial of Nonpharmacologic Intervention in the Elderly (TONE) study demonstrated significant weight loss (3.5-4.5 kg average reduction) among adults age 60-80 years over a 30-month follow-up period [53]. Similarly, initial 1-year results from the Look AHEAD (Action for Health in Diabetes) trial have shown that older adults (> 65 years) attend more lifestyle intervention sessions and participate in more physical activity than their younger counterparts [54]. At the end of a 6-month follow-up period, participants in The Weight Loss Maintenance trial demonstrated significant weight loss across racial and gender groups; weight loss was greatest among non-African American men and least among African American women [55].

Although there have been few behavioral weight loss intervention trials among persons with serious mental illness, previous work suggests that short-term weight loss can be achieved in this population [51,56]. The magnitude of weight change in ACHIEVE and other trials for persons with serious mental illness could be lower than seen in other studies and populations. If true, this may be due in part to participants having difficulty incorporating targeted behaviors from weight management sessions or lacking resources to buy lower-calorie foods. Other barriers to weight loss may include persistent mental health symptoms and frequent hospitalizations. However, the ACHIEVE Trial is unique in that interventionists provide frequent and extended contacts at locations participants regularly attend. In contrast, previous lifestyle interventions in populations without mental illness often have less frequent in-person interaction and require participants to go to other locations for intervention groups and data collection. The frequent contacts in a familiar setting in ACHIEVE may help overcome barriers from cognitive limitations and/or mental health symptoms and subsequently foster significant weight loss. The multiple components of the intervention are designed to include a variety of methods to induce behavior change through repetitive and on-going activities (e.g., group and individual sessions, rewards, food models, daily record trackers).

The ACHIEVE Trial is one of the first weight loss trials that incorporates tailored weight management sessions and on-site exercise classes to persons with serious mental illness. This multi-site study will include a diversity of racial/ethnic groups, suburban and urban areas across Maryland, younger and older adults, and persons with varying severity and types of psychiatric disease. Thus, the results should be applicable to a wide range of persons with serious mental illness.

One challenge of the ACHIEVE Trial is the extensive support and buy-in from staff at the psychiatric rehabilitation centers required for success. ACHIEVE interventionists and data collectors need the center's physical space and other resources such as time to consult with staff in order to implement the intervention and collect data. Even with enthusiasm from psychiatric rehabilitation programs, intervention implementation may still be challenging because of certain program constraints. In the current funding environment, many mental health programs are under significant financial stress and have high staff turnover.

If the ACHIEVE intervention proves effective, there will be strong justification for mechanisms to sustain the program at current sites and disseminate it to other centers. Resource data collected during the trial will inform future costs of continuing the intervention. Practical considerations for intervention sustainability and dissemination are complex and include how cardiovascular disease prevention fits into centers' priorities and what funding the rehabilitation programs would have to conduct the intervention. Centers likely would need dedicated resources or reimbursement mechanisms to contract with experienced interventionists and/or to invest in training psychiatric rehabilitation staff to conduct appropriate intervention components.

The ACHIEVE Trial tests an evidence-based approach to the problem of obesity in persons with serious mental illness. The study will provide knowledge about how to accomplish weight loss through an appropriately tailored intervention delivered in a psychiatric rehabilitation setting. Furthermore, the results from this study will inform future work in healthy lifestyle interventions for cardiovascular disease prevention in populations with chronic mental illness.

## Abbreviations

CES-D: refers to Center for Epidemiologic Studies Depression Scale; SF-12: refers to Short Form 12 Health Survey; HIPAA: refers to Health Insurance Portability and Accountability Act; GEE: refers to Generalized estimating equation.

## Competing interests

The authors declare that they have no competing interests.

## Authors' contributions

GLD conceived the design of the study. EG and NYW participated in the analytic and statistical analysis plans. KDF participated in the cost-analysis plans. GJJ participated in developing the exercise intervention and bike test measures. FBD participated in the study design. CAA, JVG, SSS, AD participated in the design and implementation of the environmental nutrition intervention. RWG participated in the intervention design. JF designed the data entry and documentation system. MO led the intervention staff. OF and LC directed data collection. JBC participated in the coordination of the trial. SSC, GLD, and LJA drafted the article. All authors edited and approved the final manuscript.

## Pre-publication history

The pre-publication history for this paper can be accessed here:

http://www.biomedcentral.com/1471-244X/10/108/prepub
